# Supplementary material for: From Real-World Data to Causally Interpretable Models: A Bayesian Network to Predict Cardiovascular Diseases in Adolescents and Young Adults with Breast Cancer
Source: Cancers (Basel). 2024 Oct 29;16(21):3643. doi: 10.3390/cancers16213643 (PMC11544965; doi:10.3390/cancers16213643)

Supplementary Material

Table S1. Algorithms used to retrieve patients' information in the administrative databases

		Administrative databases			
		Hospital discharge records		Outpatient services	Pharmaceutical flow
**Category**	**Variable**	**ICD-9-CM diagnosis codes (searched among all the 6 diagnosis)**	**ICD-9-CM procedural codes (searched among all the 6 procedures)**	**ICD-9-CM procedural codes**	**ATC codes**
Treatments	Surgery		Conservative= 8520, 8521, 8522; Radical=8523, 8533, 8534, 8535, 8536, 8541, 8542, 8543, 8544, 8545, 8546, 8547, 8548		
	Chemotherapy		V581, 9925, 9928, 9929	9925, 9928, 9929, MAC01, MAC02, MAC03, MAC04	L01A*, L01B*, L01C*, L01D*, L01E*, L01XA*, L01XB*, L01XD*, L01XF*, L01XG*, L01XH*, L01XJ*, L01XK*, L01XX*, L01XY*
	Radiotherapy		V580, 922*,923*	922*,923*	
	Target therapy				L01XC*
	Hormone therapy				L02*
Major cardio-vascular risk factors	Dyslipidemia				C10*
	Type 2 diabetes				A10*
	Hypertension				C02*, C03*, C04*, C07*, C08*, C09*

Cardio-vascular diseases	Cardiotoxicity	39891, 4260, 42610, 42611, 42612, 42613, 4262, 4263, 4264, 42650, 42651, 42652, 42653, 42654, 4266, 4267, 42681, 42682, 42689, 4269, V450, V4500, V4501, V4502, V4509, V533, V5331, V5332, V5339, 4270, 4271, 4272, 42731, 42732, 42741, 42742, 4275, 42760, 42761, 42769, 42781, 42789, 4279, 4280, 4281, 42820, 42821, 42822, 42823, 42830, 42831, 42832, 42833, 42840, 42841, 42842, 42843, 4289, 7850, 7851,			C01BC03, C01CA24, C01DA14, C08DB01, C01BC04, C01BD01, C01BD07, C03DA03, C03DA02, C07AA07, C07AA12
	Ischemic heart disease	4100, 41000, 41001, 41002, 4101, 41010, 41011, 41012, 4102, 41020, 41021, 41022, 4103, 41030, 41031, 41032, 4104, 41040, 41041, 41042, 4105, 41050, 41051, 41052, 4106, 41060, 41061, 41062, 4107, 41070, 41071, 41072, 4108, 41080, 41081, 41082, 4109, 41090, 41091, 41092, 4110, 4111, 4118, 41181, 41189, 412, 4130, 4131, 4139, 4140, 41400, 41401, 41406, 4142, 4143, 4144, 4148, 4149, V4581, V4582, 78650, 78651, 78659			
Target variable= Cardio-vascular diseases	Valvulopathies	3940, 3941, 3942, 3949, 3950, 3951, 3952, 3959, 3960, 3961, 3962, 3963, 3968, 3969, 3970, 3971, 3979, 4240, 4241, 4242, 4243, 42490, 42491, 42499, 7852, 7853, V422, V433			

	Ischemic diseases (including pulmonary embolism and pathology of the right heart and cerebrovascular events)	34660, 34661, 34662, 34663, 4100, 41000, 41001, 41002, 4101, 41010, 41011, 41012, 4102, 41020, 41021, 41022, 4103, 41030, 41031, 41032, 4104, 41040, 41041, 41042, 4105, 41050, 41051, 41052, 4106, 41060, 41061, 41062, 4107, 41070, 41071, 41072, 4108, 41080, 41081, 41082, 4109, 41090, 41091, 41092, 4110, 4111, 4118, 41181, 41189, 412, 4130, 4131, 4139, 4140, 41400, 41401, 41406, , 41410, 41411, 41412, 41419, 4142, 4143, 4144, 4148, 4149, 4150, 4151, 41512, 41513, 41519, 4160, 4161, 4162, 4168, 4169, 4170, 4171, 4178, 4179, 4291, 4292, 4293, 4295, 4296, 42971, 42979, 42981, 42982, 42983, 42989, 4299, 430, 431, 4320, 4321, 4329, 43301, 43311, 43321, 43331, 43381, 43391, 4340, 43400, 43401, 4341, 43410, 43411, 4349, 43490, 43491, 436, 4330, 43300, 4331, 43310, 4332, 43320, 4333, 43330, 4338, 43380, 4339, 43390, 4370, 4371, 4373, 4374, 4375, 4376, 4377, 4378, 4379, 4350, 4351, 4352, 4353, 4358, 4359, 438, 4380, 43810, 43811, 43812, 43813, 43814, 43819, 43820, 43821, 43822, 43830, 43831, 43832, 43840, 43841, 43842, 43850, 43851, 43852, V1255, V4581, V4582, 78650, 78651, 78659			
	Conduction disorders and arrhythmias	4260, 42610, 42611, 42612, 42613, 4262, 4263, 4264, 42650, 42651, 42652, 42653, 42654, 4266, 4267, 42681, 42682, 42689, 4269, V450, V4500, V4501, V4502, V4509, V533, V5331, V5332, V5339, 4270, 4271, 4272, 42731, 42732,			C01BC03, C01CA24, C01DA14, C08DB01, C01BC04, C01BD01,

		42760, 42761, 42769, 42781, 42789, 4279, 7850, 7851, 42741, 42742, 4275;			C01BD07, C07AA07, C07AA12
	Heart failure	39891, 4280, 4281, 42820, 42821, 42822, 42823, 42830, 42831, 42832, 42833, 42840, 42841, 42842, 42843, 4289;			C03DA03, C03DA02
	Myocarditis	03282, 03640, 03641, 03642, 03643, 07420, 07421, 07422, 07423, 11281, 11503, 11504, 11513, 11514, 11593, 11594, 1303, 3910, 3911, 3912, 3918, 3919, 3920, 393, 3980, 39890, 39899, 4200, 42090, 42091, 42099, 4210, 4211, 4219, 4220, 42290, 42291, 42292, 42293, 42299, 4230, 4231, 4232, 4233, 4238, 4239, 4250, 4251, 42511, 42518, 4252, 4253, 4254, 4257, 4258, 4259, 4290			

ICD-9-CM=International Classification of Diseases, 9th Revision, Clinical Modification
ATC=Anatomical Therapeutic Chemical codes
**= all subsequent codes must be included*

Table S2: Absolute (N) and relative (%) distribution of the patients included in the analysis, by cohort type.

		**Population-based cohort (PBC)**	**Clinical-based cohort (CBC)**
		Total=1036 cases	Total=339 cases
		N (%)	N (%)
Age at diagnosis	18-34	336 (33%)	123 (36%)
	35-39	700 (67%)	216 (64%)
Grading	I	0 (0%)	13 (4%)
	II	0 (0%)	115 (34%)
	III	0 (0%)	173 (51%)
	Missing	1036 (100%)	38 (11%)
Vascular invasion	No	0 (0%)	101 (30%)
	Yes	0 (0%)	92 (27%)
	Missing	1036 (100%)	146 (43%)
Ki67+	No	0 (0%)	25 (7%)
	Yes	0 (0%)	287 (85%)
	Missing	1036 (100%)	27 (8%)
Primary tumor receptor status	HER 2 enriched	0 (0%)	29 (9%)
	Luminal	0 (0%)	3 (1%)
	Luminal A	0 (0%)	25 (7%)
	Luminal B	0 (0%)	134 (40%)
	Luminal HER2	0 (0%)	64 (19%)
	Triple negative	0 (0%)	64 (19%)
	Missing	1036 (100%)	20 (6%)
Pathological tumor size	pT1	0 (0%)	163 (48%)
	pT2	0 (0%)	46 (14%)
	pT3	0 (0%)	3 (1%)
	pT4	0 (0%)	7 (2%)
	Missing	1036 (100%)	120 (35%)
Lymph node involvement	pN+	0 (0%)	90 (27%)
	pN0	0 (0%)	140 (41%)
	Missing	1036 (100%)	109 (32%)

Histology	Ductal and lobular neoplasm	931 (90%)	280 (83%)
	Epithelial neoplasms, NOS	39 (4%)	0 (0%)
	Adenocarcinomas	22 (2%)	30 (9%)
	Neoplasms, NOS	27 (2%)	0 (0%)
	Other histologies	17 (2%)	17 (5%)
	Missing	0 (0%)	12 (3%)
Surgery	Conservative	603 (58%)	128 (38%)
	Radical	433 (42%)	211 (62%)
Treatment	Surgery + Adjuvant	749 (72%)	229 (67%)
	Neoadjuvant + surgery + adjuvant	220 (21%)	74 (22%)
	Surgery only	52 (5%)	0 (0%)
	Neoadjuvant + surgery	15 (2%)	24 (7%)
	Incomplete information	0 (0%)	12 (4%)
Dyslipidemia	Post-treatment	32 (4%)	0 (0%)
	Pre-treatment	15 (1%)	17 (5%)
	No	989 (95%)	0 (0%)
	Missing	0 (0%)	322 (95%)
Type 2 diabetes	Post-treatment	15 (1%)	0 (0%)
	Pre-treatment	3 (0%)	2 (1%)
	No	1018 (99%)	0 (0%)
	Missing	0 (0%)	337 (99%)
Hypertension	Post-treatment	181 (17%)	0 (0%)
	Pre-treatment	37 (4%)	11 (3%)
	No	818 (79%)	0 (0%)
	Missing	0 (0%)	328 (97%)
Cardiovascular diseases	Cardiotoxicities	23 (2%)	0 (0%)
	Ischemic heart diseases	5 (0%)	0 (0%)
	Other	12 (1%)	0 (0%)
	None	996 (97%)	0 (0%)
	Missing	0 (0%)	339 (100%)

Death in 5 years	No	914 (88%)	151 (44%)
	Yes	18 (2%)	2 (1%)
	Missing	104 (10%)	186 (55%)

Figure S1: Flow-chart followed for the model development

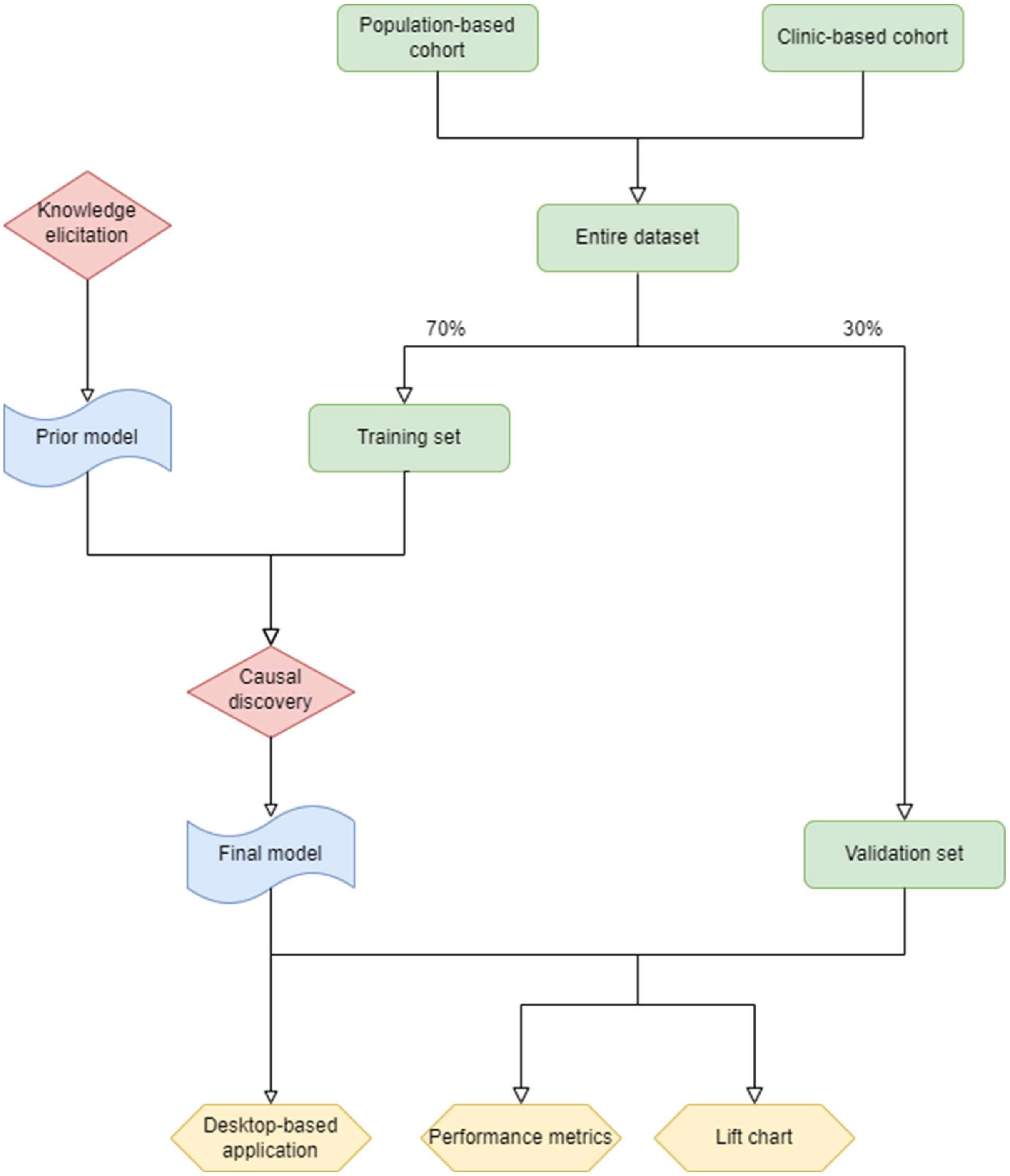

Supplement: Supplementary file 1 [file cancers-16-03643-s001.zip › cancers-3232586-supplementary.pdf]
